# Individual and joint association of serum 25-hydroxyvitamin D and folate levels with the risk of sarcopenia: a cross-sectional study from the NHANES 2011–2018

**DOI:** 10.3389/fnut.2025.1576705

**Published:** 2025-06-23

**Authors:** Yaping Wei, Qiangqiang He, Qiannan Di, Jing Li, Jingyi Zhang, Lixin Na

**Affiliations:** ^1^College of Public Health, Shanghai University of Medicine and Health Sciences, Shanghai, China; ^2^Tsinghua Shenzhen International Graduate School, Tsinghua University, Shenzhen, China

**Keywords:** 25(OH)D, folate, sarcopenia, joint association, cross-sectional study

## Abstract

**Background:**

Recent studies have indicated that vitamin D and folate are essential for muscle health and each is independently linked to the prevalence of sarcopenia. However, the potential synergistic effects of vitamin D and folate on sarcopenia have not been extensively studied. This study aims to investigate both the individual and joint associations of serum 25-hydroxyvitamin D [25(OH)D] and folate concentrations with the risk of sarcopenia.

**Methods:**

This study conducted a cross-sectional analysis using data from the National Health and Nutrition Examination Survey (NHANES), covering the years 2011–2018. Multivariable logistic regression models were used to determine individual and joint associations of serum 25(OH)D and folate with sarcopenia. Additionally, the relative excess risk due to interaction (RERI) was estimated to assess additivity.

**Results:**

A significant inverse relationship was observed between serum 25(OH)D and folate levels in relation to sarcopenia. Specifically, among participants with 25(OH)D levels < 50 nmol/L, the odds ratios (ORs) for sarcopenia were 0.67 [95% confidence interval (CI): 0.56–0.81] for those with 25(OH)D levels between 50 and 75 nmol/L, and 0.61 (95% CI: 0.48, 0.77) for those with levels > 75 nmol/L. Similarly, when comparing participants in the lowest tertile of folate, the ORs for sarcopenia were 0.74 (95% CI: 0.61, 0.89) for the second tertile and 0.72 (95% CI: 0.59, 0.88) for the third tertile. Among individuals with fasting blood glucose levels < 7.0 mmol/L, those with both low levels of 25(OH)D and folate exhibited a significantly higher risk of sarcopenia compared to those with high levels of 25(OH)D and folate. Furthermore, the RERI was statistically significant.

**Conclusion:**

In the present study, a synergistic interaction between 25(OH)D and folate was observed in relation to the risk of sarcopenia. These findings contribute new insights into the nutritional factors associated with sarcopenia and pave the way for future longitudinal studies to further explore this association.

## 1 Introduction

Sarcopenia is a growing geriatric syndrome characterized by the loss of muscle mass and strength, affecting approximately 20.7% of older adults in China (1). Previous studies have identified several key risk factors associated with the development of sarcopenia, including age, nutritional deficiencies, and chronic diseases. These factors not only contribute to the onset of sarcopenia but also exacerbate its progression, leading to severe health consequences such as increased disability, hospitalization, and mortality rates among affected individuals (2). Among some nutrients linked to muscle health, Vitamin D and folate have garnered attention due to their essential roles in a range of physiological processes. Vitamin D, especially in its active form 25-hydroxyvitamin D [25(OH)D], is crucial for maintaining bone health, modulating immune responses, and supporting muscle function. Studies have shown that adequate levels of 25(OH)D are associated with improved muscle strength and mass, suggesting a protective role against sarcopenia (3). Folate is essential for DNA synthesis and repair, which are vital processes for muscle cell proliferation and regeneration (4). Therefore, folate may also be involved in the onset and progression of sarcopenia.

Recent studies have highlighted the potential link between vitamin D and folate deficiencies and the increased risk of sarcopenia (5). Research conducted on aging populations in both Northern and Southern Italy has found a significant association between serum deficiencies in vitamin D and folate and the likelihood of developing sarcopenia (6). Epidemiological studies showed clinical correlations between vitamin D deficiency and sarcopenia, which has led to growing attention within the scientific community. In the general population, low serum vitamin D concentrations have been strongly associated with a higher prevalence of sarcopenia and a decline in physical performance, such as walking speed (7). Several meta-analyses and randomized controlled trials (RCTs) have further demonstrated that vitamin D supplementation can improve overall muscle strength, particularly in the lower limbs, and physical function (8, 9). Folate deficiency has also been found to negatively impact muscle strength. Recent studies indicate that lower serum folate levels are significantly correlated with a reduction in leg and grip strength among older adults, especially those over 65 years of age with diabetes mellitus, with a more pronounced effect in women (10). Furthermore, reduced dietary intake of folate and other essential nutrients has been shown to impair oxidative capacity, which negatively affects muscle health, as demonstrated in studies involving aged mice (11). However, despite these finding, the potential synergistic effects of vitamin D and folate in relation to sarcopenia remain largely unexplored.

This study aims to investigate the relationship between serum 25(OH)D and folate concentrations and the risk of sarcopenia by analyzing data from the National Health and Nutrition Examination Survey (NHANES). By examining the roles of these two key nutrients, this research seeks to clarify their individual and combined contributions to sarcopenia, thereby enhancing our understanding of the nutritional factors that influence muscle health. Given the increasing prevalence of sarcopenia in aging societies, understanding the interplay between vitamin D, folate, and sarcopenia is crucial for developing effective strategies to prevent and treat this condition. The results of this study could provide valuable insights for designing both preventive measures and therapeutic approaches to combat sarcopenia.

## 2 Materials and methods

### 2.1 Study population

This study utilized data from the NHANES conducted between 2011 and 2018. NHANES is a cross-sectional study designed to assess the health and nutritional status of the U.S. population. It employs a complex, multistage probability sampling method to ensure a representative sample of the civilian, non-institutionalized population. Data are collected through in-home interviews, physical examinations, and biospecimen collection, with approximately 5,000 individuals assessed each year. For this study, we focused on a cohort of 22,617 individuals aged 20 years and older. Participants with incomplete data on sarcopenia or missing measurements of 25(OH)D or folate concentrations were excluded. As a result, 9,489 participants were included in the final analysis (Supplementary Figure 1).

### 2.2 Laboratory measurements

Blood samples were collected from participants after an overnight fast. To assess body composition, dual-energy X-ray absorptiometry (DXA) was used, which is known for its speed, ease of use, and minimal radiation exposure. DXA provided comprehensive bone and soft tissue measurements for the entire body, including both arms and legs. Serum 25(OH)D levels were analyzed using liquid chromatography-tandem mass spectrometry (LC-MS/MS), while serum total folate was measured with isotope-dilution high-performance LC-MS/MS. Blood glucose concentrations were quantified using the oxygen rate method with a Beckman oxygen electrode, which employs the glucose oxidase technique. Serum cholesterol, triglyceride levels, and uric acid concentrations were assessed with the DxC800 analyzer using a timed-endpoint method. Additionally, the DxC800 was used to measure AST activity in serum or plasma via an enzymatic rate method and ALT activity using a kinetic rate method. Finally, serum creatinine concentrations were determined with the Jaffe rate method, based on kinetic alkaline picrate, on the DxC800 modular chemistry system.

### 2.3 Covariate assessment

Demographic and health-related data, including age, gender, race, monthly family income, educational attainment, month of examination, smoking status, alcohol use, and diagnoses of hypertension, diabetes, and setting time, were obtained through face-to-face interviews. Trained personnel measured participants’ height, weight, and waist circumference. The triglyceride-glucose (TyG) index, calculated as TyG = Ln [fasting triglyceride (mg/dl) × fasting plasma glucose (mg/dl)/2], was used as an indicator of insulin sensitivity. Appendicular skeletal muscle mass (ASM) was normalized by dividing it by body mass index (BMI) to further assess sarcopenia. Since the study population consisted of U.S. adults from NHANES, we applied the National Institutes of Health (NIH) criteria for sarcopenia, which define it as an ASM/BMI ratio of less than 0.789 for men and less than 0.512 for women. The ASM/BMI thresholds were established by The Foundation for the National Institutes of Health Biomarkers Consortium Sarcopenia Project, which used an evidence-based approach to develop these criteria (12). By dividing ASM by BMI, we can mitigate the influence of body weight on the absolute value of lean body mass.

### 2.4 Statistical analysis

Continuous variables were presented as mean ± SD and compared using parametric *t* tests. Categorical variables were expressed as counts and percentages and compared using Chi-square tests. All covariates had less than 5% missing data. Missing values for continuous variables were replaced with the median, while missing values for categorical variables were replaced by the most frequent category. We estimated both the individual and joint associations of serum 25(OH)D and folate with sarcopenia using logistic regression models, calculating odds ratios (ORs) and 95% confidence intervals (CIs) for sarcopenia. Potential confounders were selected on variables commonly reported in the literature and those showing significant associations (*P* < 0.05) in univariate analyses. In the models examining the joint effects of serum 25(OH)D and folate, binary categorizations were used, with serum 25(OH)D levels of ≥50 nmol/L and folate levels ≥ the median serving as the reference group.

To assess additive interaction, we calculated the relative excess risk due to interaction (RERI) using the formula:

RERI = HR (++) − HR (+−) − HR (−+) + 1.

We also computed the attributable proportion (AP) as AP = RERI/HR++, considering both RERI and AP significant if their 95% CIs did not include zero. Additionally, the synergy index (S) was calculated using the formula:

S = [OR (++) − 1] / [OR (+−) + OR (−+)].

With significance determined when the 95% CIs did not include one. Statistical significance was determined using two-tailed tests with a significance threshold of 0.05. All statistical analyses were performed using R software, version 3.2.5.^1^

## 3 Results

### 3.1 Study participants and baseline characteristics

Table 1 presents the baseline characteristics of the participants, stratified by sarcopenia status. The mean age of the participants was 39.1 years, and 52.5% of the total sample was female. Compared with non-sarcopenic participants, participants with sarcopenia were older and more likely to be female, of Mexican American descent, have an education level below the 9th grade, and be non-smokers and non-drinkers. In addition, they had higher values in BMI, waist circumference, cholesterol, triglycerides, glucose, and setting time, but lower levels of creatinine, 25(OH)D, intake of energy, protein, carbohydrate, and total fat. Our analysis revealed significant differences between included (*n* = 9,489) and excluded (*n* = 29,667) participants across multiple baseline variables (Supplementary Table 1), with excluded participants tending to be younger, more likely to be male and of Mexican American ethnicity, having lower educational level, higher rates of missing data on smoking and alcohol use, lower prevalence of hypertension and diabetes, longer sedentary time, lower BMI and waist circumference measurements, more favorable lipid profiles (lower total cholesterol and triglycerides), elevated levels of serum creatinine, blood glucose, folate and 25(OH)D, and lower level of energy and macronutrients consuming compared to the included persons.

**TABLE 1:** Baseline and clinical characteristics of the study population by sarcopenia status.

Variables	Total	Non-sarcopenia	Sarcopenia	*P*
n	9,489	8,729	760	
Age, years	39.1 ± 11.4	38.6 ± 11.3	44.0 ± 11.1	<0.001
Gender				0.009
Male	4,508 (47.5)	4,182 (47.9)	326 (42.9)	
Female	4,981 (52.5)	4,547 (52.1)	434 (57.1)	
Race				<0.001
Mexican American	1,440 (15.2)	1,140 (13.1)	300 (39.5)	
Non-Hispanic White	990 (10.4)	878 (10.1)	112 (14.7)	
Non-Hispanic Black	3,377 (35.6)	3,175 (36.4)	202 (26.6)	
Non-Hispanic Asian	1,916 (20.2)	1,874 (21.5)	42 (5.5)	
Other Race	1,766 (18.6)	1,662 (19.0)	104 (13.7)	
Education level				<0.001
Less than 9th grade	581 (6.1)	443 (5.1)	138 (18.2)	
9th–11th grade	1,139 (12.0)	1,013 (11.6)	126 (16.6)	
High school graduate	2,041 (21.5)	1,853 (21.2)	188 (24.7)	
Some college	3,118 (32.9)	2,914 (33.4)	204 (26.8)	
College graduate or above	2,608 (27.5)	2,504 (28.7)	104 (13.7)	
Season of examination				<0.001
November 1 through April 30	4,644 (48.9)	4,201 (48.1)	443 (58.3)	
May 1 through October 31	4,845 (51.1)	4,528 (51.9)	317 (41.7)	
Smoking				<0.001
No	2,031 (21.4)	1,843 (21.1)	188 (24.7)	
Yes	1,674 (17.6)	1,590 (18.2)	84 (11.1)	
Missing	5,784 (61.0)	5,296 (60.7)	488 (64.2)	
Alcohol				<0.001
No	1,872 (19.7)	1,654 (18.9)	218 (28.7)	
Yes	5,692 (60.0)	5,301 (60.7)	391 (51.4)	
Missing	1,925 (20.3)	1,774 (20.3)	151 (19.9)	
Hypertension				<0.001
Yes	2,187 (23.0)	1,938 (22.2)	249 (32.8)	
No	7,302 (77.0)	6,791 (77.8)	511 (67.2)	
Diabetes				<0.001
Yes	685 (7.2)	566 (6.5)	119 (15.7)	
No	8,635 (91.0)	8,017 (91.9)	612 (80.6)	
Borderline	169 (1.8)	141 (1.6)	28 (3.7)	
Setting time, min	376.0 ± 204.1	377.8 ± 203.4	355.8 ± 211.6	0.004
Body mass index, kg/m^2^	28.8 ± 6.8	28.3 ± 6.5	34.3 ± 7.9	<0.001
Waist circumference, cm	96.9 ± 16.3	96.0 ± 15.9	108.2 ± 17.0	<0.001
Cholesterol, mmol/L	4.9 ± 1.0	4.9 ± 1.0	5.1 ± 1.1	<0.001
Triglyceride, mmol/L	1.7 ± 1.7	1.7 ± 1.7	2.0 ± 1.4	<0.001
Creatinine, μmol/L	74.9 ± 32.6	75.5 ± 32.1	67.3 ± 36.4	<0.001
Glucose, mmol/L	5.5 ± 2.1	5.4 ± 2.0	6.2 ± 2.8	<0.001
Folate, nmol/L	38.5 ± 22.8	38.5 ± 23.0	37.9 ± 20.4	0.445
25(OH)D, nmol/L	60.4 ± 24.9	60.7 ± 25.0	57.1 ± 23.7	<0.001
Intake of energy (kcal/day)	2,229.1 ± 1,011.9	2,249.0 ± 1,022.0	2,000.4 ± 856.3	<0.001
Intake of protein (g/day)	85.8 ± 44.1	86.4 ± 44.5	78.8 ± 37.8	<0.001
Intake of carbohydrate (g/day)	265.5 ± 128.1	267.3 ± 129.2	244.7 ± 113.0	<0.001
Intake of total fat (g/day)	85.3 ± 47.8	86.2 ± 48.3	75.0 ± 40.1	<0.001

### 3.2 Individual associations of 25(OH)D and folate concentrations with sarcopenia

Table 2 presents the results from multivariable logistic regression models examining the association between 25(OH)D, folate, and sarcopenia. Continuous 25(OH)D levels were significantly associated with a higher risk of sarcopenia both before and after adjusting for potential confounders. To categorize 25(OH)D levels, clinically established cutoff values were used. Compared to participants with 25(OH)D levels < 50 nmol/L, those with levels ranging from 50 to 75 nmol/L demonstrated a significantly reduced risk of sarcopenia (OR = 0.67, 95% CI: 0.56, 0.81, *P* < 0.001). Individuals with 25(OH)D levels ≥ 75 nmol/L exhibited an even lower risk (OR = 0.61, 95% CI: 0.48, 0.77, *P* < 0.001).

**TABLE 2:** Individual associations between 25(OH)D and folate levels with the risk of sarcopenia.

Variables	*n*	Case (%)	Crude model		Adjusted model	
			OR (95% CI)	*P*	OR (95% CI)	*P*
**25(OH)D, nmol/L**						
Per SD	9,489	760 (8.0)	0.86 (0.79, 0.93)	<0.001	0.81 (0.73, 0.89)	<0.001
<50	3,427	323 (9.4)	Ref		Ref	
50–75	3,756	289 (7.7)	0.80 (0.68, 0.95)	0.009	0.67 (0.56, 0.81)	<0.001
>75	2,306	148 (6.4)	0.66 (0.54, 0.81)	<0.001	0.61 (0.48, 0.77)	<0.001
*P* for trend				<0.001		<0.001
**Folate, nmol/L**						
Per SD	9,489	760 (8.0)	0.97 (0.89, 1.05)	0.440	0.90 (0.81,1.00)	0.040
T1 (<27.4)	3,154	274 (8.7)	Ref		Ref	
T2 (27.4–42.4)	3,154	245 (7.8)	0.89 (0.74, 1.06)	0.184	0.74 (0.61, 0.89)	0.002
T3 (≥42.4)	3,181	241 (7.6)	0.86 (0.72, 1.03)	0.106	0.72 (0.59, 0.88)	0.002
*P* for trend				0.104		0.001

The adjusted model was controlled for age, sex, race, family income, education level, the season of examination, smoking status, alcohol use, sitting time, cholesterol, triglyceride, creatinine, hypertension, diabetes, intake of energy, protein, carbohydrate, and total fat. Additionally, serum folate levels were further adjusted for 25(OH)D levels. 25(OH)D, 25-hydroxyvitamin D.

**FIGURE 1:**
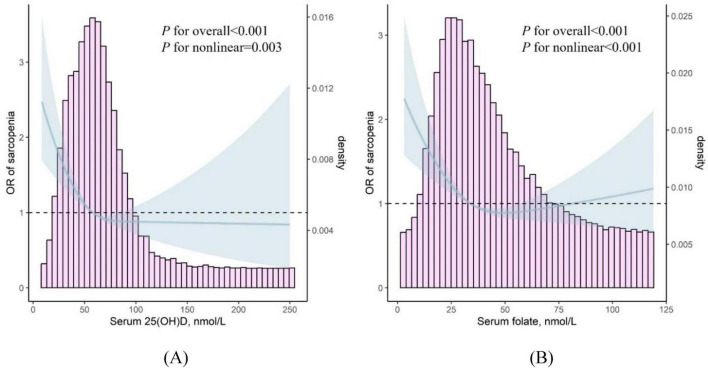
**(A)** Restricted cubic spline of the association between serum 25(OH)D levels and the risk of sarcopenia. **(B)** Restricted cubic spline of the association between serum folate and the risk of sarcopenia.

Logistic regression analysis also revealed a significant association between serum folate levels and sarcopenia risk after adjusting for confounders. Participants in the second and third tertiles of folate had a lower risk of sarcopenia compared with those in the first tertile. The ORs for sarcopenia in the second tertile (27.4–42.4 nmol/L) were 0.74 (95% CI: 0.61, 0.89, *P* = 0.002), and for the third tertile (≥42.4 nmol/L), the ORs were 0.72 (95% CI: 0.59, 0.88, *P* = 0.002). Furthermore, spline analyses revealed an L-shaped relationship between 25(OH)D and folate levels with the risk of sarcopenia, as shown in Figure 1.

We conducted additional analyses to clarify ethnic and socioeconomic influences (Supplementary Table 2). In multivariable-adjusted models incorporating race and monthly family income, the inverse association between vitamin D and sarcopenia persisted robustly. Serum folate showed a protective effect only in adjusted models, suggesting confounding by sociodemographic factors (Supplementary Table 2). Stratified analyses showed no significant interactions by race (*P* for interaction = 0.365) or monthly family income (*P* for interaction = 0.909) (Supplementary Figure 3). The inverse association between serum folate and sarcopenia was statistically significant only among Mexican Americans. A significant interaction was detected between race and baseline folate levels (*P* = 0.005). No significant interaction was observed between monthly family income and folate levels (Supplementary Figure 4). To address the potential underestimation of ASM due to BMI adjustment, we conducted sensitivity analyses excluding participants with BMI ≥ 30. The associations of vitamin D deficiency and folate deficiency with sarcopenia remained statistically significant (Supplementary Table 3).

### 3.3 Joint associations of 25(OH)D and folate with sarcopenia in the overall population and subgroups stratified by blood glucose levels

The highest OR for sarcopenia were observed among participants with 25(OH)D levels < 50 nmol/L and folate levels < 27.4 nmol/L, compared to those with 25(OH)D levels ≥ 50 nmol/L and folate levels ≥ 27.4 nmol/L. These effects were even more pronounced in individuals with serum glucose levels below 7 mmol/L. Specifically, among those with fasting blood glucose levels below 7.0 mmol/L, participants with 25(OH)D < 50 nmol/L and folate < 27.4 nmol/L had the highest risk of sarcopenia (OR = 2.38, 95% CI = 1.87, 3.03), compared to individuals with 25(OH)D ≥ 50 nmol/L and folate ≥ 27.4 nmol/L. A significant interaction was detected between baseline 25(OH)D and folate (*P* = 0.037). To assess this additive interaction, we calculated the RERI and the AP, as shown in Table 3. A statistically significant additive interaction was found, with a RERI of 0.835 (95% CI: 0.250, 1.450) between insufficient 25(OH)D levels and low folate. To further illustrate these findings, we developed a chart (Supplementary Figure 2) that depicts the combined effects of 25(OH)D and folate on sarcopenia. In this chart, the reference group consisted of individuals with higher levels of both 25(OH)D and folate. Participants with lower levels of both 25(OH)D and folate exhibited the highest odds of sarcopenia compared to the reference group.

**TABLE 3:** Joint association between 25(OH)D and folate levels with the risk of sarcopenia.

Vitamin D, nmol/L	Folate, nmol/L	*N*	Case (%)	Crude model		Adjusted model	
				OR (95% CI)	*P*	OR (95% CI)	*P*
**Total people**							
≥50	≥27.4	4,504	321 (7.1)	Ref		Ref	
≥50	<27.4	1,558	116 (7.4)	1.05 (0.84, 1.31)	0.675	1.24 (0.98, 1.57)	0.070
<50	≥27.4	1,831	165 (9.0)	1.29 (1.06, 1.57)	0.011	1.35 (1.09, 1.68)	0.006
<50	<27.4	1,596	158 (9.9)	1.43 (1.17, 1.75)	<0.001	2.07 (1.65, 2.59)	<0.001
Interaction					0.727		0.233
RERI: 0.471 (−0.050, 0.991)							
AP: 0.228 (−0.037, 0.415)							
**Glucose < 7 nmol/L**							
≥50	≥27.4	4,199	269 (6.4)	Ref		Ref	
≥50	<27.4	1,479	95 (6.4)	1.00 (0.79, 1.28)	0.982	1.16 (0.90, 1.50)	0.246
<50	≥27.4	1,646	130 (7.9)	1.25 (1.01, 1.56)	0.042	1.38 (1.09, 1.76)	0.008
<50	<27.4	1,473	145 (9.8)	1.60 (1.29, 1.97)	<0.001	2.38 (1.87, 3.03)	<0.001
Interaction					0.174		0.037
RERI: 0.835 (0.250, 1.450)							
AP: 0.350 (0.105, 0.521)							
**Glucose ≥ 7 nmol/L**							
≥50	≥27.4	312	55 (17.6)	Ref		Ref	
≥50	<27.4	72	18 (25.0)	1.56 (0.85, 2.86)	0.153	1.90 (0.96, 3.75)	0.065
<50	≥27.4	191	37 (19.4)	1.12 (0.71, 1.78)	0.624	1.06 (0.63, 1.78)	0.838
<50	<27.4	117	11 (9.4)	0.48 (0.24, 0.96)	0.039	0.65 (0.30, 1.37)	0.255
Interaction					0.015		0.050
RERI: −1.147 (−3.100, −0.0369)							
AP: −1.746 (−6.460, −0.340)							

Adjusted model was adjusted for age, sex, race, family income, education level, season of examination, smoking status, alcohol use, sitting time, cholesterol, triglyceride, creatinine, hypertension, diabetes, intake of energy, protein, carbohydrate, and total fat. AP, attributable proportion due to interaction; RERI, relative excess risk due to interaction; OR, odds ratio.

### 3.4 Inflammation as a mediator in the associations between vitamin D, folate, and sarcopenia risk

Table 4 presents the mediating effects and proportions of biomarkers related to inflammation, oxidative stress, and insulin resistance in the context of vitamin D and folate exposure, and their association with the prevalence of sarcopenia. Specifically, the levels of alkaline phosphatase and gamma-glutamyl transferase mediated the positive association between vitamin D deficiency and the risk of sarcopenia, accounting for 7.97% and 2.10% of the total effect, respectively. Additionally, the TyG index partially mediated the relationship between vitamin D deficiency and sarcopenia, contributing 5.72% to the overall effect.

**TABLE 4:** The mediation effect between vitamin D insufficient, low folate concentration and sarcopenia.

Pathways	Indirect effect	95% CI	Mediation (%)	95% CI	*P*
**Vitamin D insufficiency**					
White blood cells	7.25E-04	−3.86E-04, 0.00	2.53	−0.017, 0.07	0.200
Alkaline phosphatase	2.15E-03	1.15E-03, 0.00	7.97	0.037, 0.16	<0.001
Bilirubin	1.74E-04	−4.83E-04, 0.00	0.53	−0.018, 0.04	0.630
Gamma-glutamyl transferase	5.85E-04	1.01E-04, 0.00	2.10	0.039, 0.05	0.016
TyG index	1.71E-03	9.14E-04, 0.00	5.72	0.276, 0.11	<0.001
**Low folate level**					
White blood cells	1.82E-03	6.86E-04, 0.00	7.48	0.027, 0.16	<0.001
Alkaline phosphatase	1.70E-03	8.81E-04, 0.00	7.21	0.032, 0.16	<0.001
Bilirubin	1.84E-03	9.70E-04, 0.00	7.67	0.037, 0.18	<0.001
Gamma-glutamyl transferase	2.49E-04	−7.98E-06, 0.00	0.99	−0.00049, 0.03	0.076
TyG index	6.78E-04	9.70E-05, 0.00	2.65	0.035, 0.07	0.024

Adjusted for age, sex, race, family income, education level, season of examination, smoking status, alcohol use, sitting time, cholesterol, triglyceride, creatinine, hypertension, and diabetes, intake of energy, intake of protein, intake of carbohydrate, and intake of total fat.

Furthermore, the levels of white blood cells, alkaline phosphatase, and bilirubin significantly mediated the positive association between low folate levels and the prevalence of sarcopenia, accounting for 7.48%, 7.21%, and 7.67% of the total effect, respectively. The TyG index also partially mediated the relationship between low folate levels and sarcopenia, accounting for 2.65% of the total effect. We also performed stratified mediation analyses comparing participants with and without chronic diseases (Supplementary Tables 4, 5). Supplementary Tables 6, 7 presents the results of the regression analysis to assess the association between 25(OH)D and folate with relative mediator biomarkers.

## 4 Discussion

To the best of our knowledge, this is the first study to examine the combined association of serum 25(OH)D and folate levels with sarcopenia. Our findings reveal a negative correlation between serum levels of 25(OH)D and folate and the risk of sarcopenia in the general U.S. population. Additionally, we observed synergistic interactions between 25(OH)D and folate in relation to sarcopenia. Furthermore, inflammation and insulin resistance were identified as contributing factors that help explain the relationship between serum vitamin D and folate levels and the risk of sarcopenia.

Consistent with multiple studies, our research identified a negative association between sarcopenia risk and serum 25(OH)D. A Mendelian randomization analysis supported this finding, showed that lower serum 25(OH)D levels are associated with an increased risk of sarcopenia, particularly in individuals with suboptimal vitamin D levels (13). In addition, a comparative analysis revealed that bioavailable 25(OH)D levels were significantly lower in sarcopenic patients compared to non-sarcopenic individuals, suggesting that vitamin D status plays a crucial role in muscle health (14). Similarly, a study focusing on postmenopausal women found that vitamin D deficiency was linked to reduced muscle strength and physical performance, further reinforcing the idea that adequate vitamin D levels are essential for maintaining muscle function (15). Another investigation of older adults highlighted a positive association between serum 25(OH)D levels and muscle strength, particularly in men, suggesting a potential sex-dependent relationship in the context of sarcopenia (16). Furthermore, research has shown that vitamin D supplementation can enhance muscle strength and function in older adults, indicating that addressing vitamin D deficiency may be an effective strategy for preventing or mitigating sarcopenia (17). Finally, a comprehensive review of epidemiological evidence emphasized the high prevalence of vitamin D deficiency among the elderly and its association with various musculoskeletal conditions, including sarcopenia, underscoring the importance of monitoring and managing vitamin D levels in this population (18). Taken together, these findings reinforce the hypothesis that maintaining adequate 25(OH)D levels are crucial for preserving muscle mass and function, particularly in older adults at higher risk for sarcopenia.

As a key component in one-carbon metabolism, serum folate has been inversely associated with sarcopenia, consistent with findings from previous studies, including the Salus in Apulia Study (6) and a case-control study conducted in Singapore (10). A meta-analysis (5) also supports this association, indicating that low folate intake is linked to an increased risk of sarcopenia. Furthermore, Zhang et al. demonstrated that higher serum folate levels are positively correlated with increased grip strength in females (19). In addition, studies suggest that dietary factors—particularly the inflammatory potential of the diet, with a specific focus on folate—may influence sarcopenia risk, underscoring the role of nutritional interventions in managing inflammation and maintaining muscle health (20). Folate deficiency has been closely associated with elevated serum homocysteine (Hcy) levels. And recent studies indicate that up to 50% of individuals over the age of 60 have elevated Hcy concentrations (21). Elevated Hcy levels have been identified as an independent risk factor for fractures (22). Moreover, studies using Mendelian randomization have found that higher circulating Hcy levels correlate with reduced grip strength, slower walking speed, and lower limb muscle mass (23).

Nevertheless, there is insufficient data regarding the joint association between serum 25(OH)D and folate and the onset of sarcopenia. The results of this study showed that low levels of both 25(OH)D and folate are associated with a higher risk compared to when these nutrients are considered individually. This finding underscores the importance of considering the interplay between different nutrients when evaluating their impact on health outcomes. The synergistic effects of vitamin D and folate in reducing sarcopenia risk may be closely linked to their roles in modulating inflammatory responses and insulin resistance. Both vitamin D and folate help regulate the secretion of inflammatory cytokines, thereby reducing chronic low-grade inflammation, a key factor in the development of sarcopenia (24, 25). Furthermore, folate deficiency can lead to elevated plasma homocysteine (Hcy) levels, which are also implicated in sarcopenia. One proposed mechanism is that high Hcy levels activate inflammatory pathways, inducing a pro-inflammatory state that promotes the secretion of cytokines. These cytokines, in turn, impair muscle regeneration and contribute to muscle wasting.

Skeletal muscle plays a crucial role as a primary target organ for insulin, significantly influencing glucose metabolism and overall energy homeostasis. Insulin resistance, a condition in which the body’s cells become less responsive to insulin, is a key factor contributing to various metabolic disorders, including type 2 diabetes and sarcopenia (muscle wasting) (26). Studies have shown that vitamin D deficiency is strongly associated with insulin resistance, and supplementing with vitamin D can improve insulin sensitivity. This suggests that vitamin D may play a positive role in preventing metabolic syndrome and diabetes (27). Similarly, folate, an important vitamin, has also been linked to insulin resistance and metabolic syndrome. A meta-analysis found that folate supplementation can significantly reduce indicators of insulin resistance, such as HOMA-IR, indicating its potential to improve insulin sensitivity (28). Furthermore, studies have shown that vitamin D and folic acid can promote Akt activation, which in turn activates the mTOR (mechanistic target of rapamycin) pathway. This pathway enhances muscle synthesis and inhibits muscle protein degradation (4, 29). This finding offer valuable insights for clinical practice, especially in nutritional interventions targeting the elderly population. The synergistic effects of vitamin D and folic acid could lead to improved health outcomes, highlighting their potential benefits in managing insulin resistance and promoting muscle health.

The observed stronger association between low serum 25(OH)D and folate levels with sarcopenia in individuals with fasting glucose < 7 mmol/L may indicate a unique nutrient-glucose interaction that contributes to early metabolic dysregulation. In contrast, in individuals with fasting glucose ≥ 7 mmol/L, the accumulation of advanced glycation end products (AGEs) and chronic inflammatory primarily drive muscle catabolism, which could obscure the independent effects of vitamin D and folate deficiencies on muscle mass (30). Moreover, in individuals with normal glucose regulation or early-stage dysglycemia, subclinical nutritional deficiencies may further exacerbate metabolic disturbances by disrupting glucose homeostasis.

This study has several limitations. Firstly, while our models were adjusted for potential confounders, the cross-sectional nature of NHANES data fundamentally precludes determining temporal sequence. Specifically, low 25(OH)D and folate levels could be both a cause and consequence of sarcopenia. For instance, reduced mobility in sarcopenic individuals may lead to decreased sunlight exposure and altered dietary intake, creating reverse causation bias. Secondly, the lack of data on diabetes duration, medication use, and glycemic control may introduce unmeasured confounding, potentially leading to overestimation of micronutrient effects in subgroup analyses. Future prospective studies incorporating detailed metabolic profiling would help clarify these interactions. Additionally, the study’s focus on American adults may limit the generalizability of the results to other populations. Furthermore, our reliance on serum folate as a composite measure of total exposure (diet, supplements, and fortified foods), without data on specific sources, limits mechanistic interpretation. Although seasonal variation in blood collection was statistically adjusted and standardized protocols were implemented, the single time-point measurements of 25(OH)D and folate may not fully reflect long-term nutritional status. Future studies that incorporate repeated measures across multiple seasons would provide a more accurate representation of true nutritional states. For future investigations, multicenter longitudinal cohorts should incorporate serial measurements of serum 25(OH)D and folate concentrations alongside standardized sarcopenia assessments to elucidate temporal relationships between nutrient trajectories and sarcopenia.

## 5 Conclusion

In conclusion, the findings of this study suggest an inverse correlation between serum 25(OH)D and folate levels and the risk of sarcopenia, with inflammatory responses and insulin resistance serving as partial mediators. Additionally, our analysis indicates that vitamin D and folate work synergistically in the development of sarcopenia. These insights open new possibilities for the prevention and treatment of sarcopenia, positioning vitamin D and folate as potential therapeutic targets.

## Data Availability

The raw data supporting the conclusions of this article will be made available by the authors, without undue reservation.

## Footnotes

1
http://www.R-project.org/
