# Supplementary material for: Individual and joint association of serum 25-hydroxyvitamin D and folate levels with the risk of sarcopenia: a cross-sectional study from the NHANES 2011–2018
Source: Front Nutr. 2025 Jun 23;12:1576705. doi: 10.3389/fnut.2025.1576705 (PMC12229844; doi:10.3389/fnut.2025.1576705)
Supplement: Supplementary file 1 [file Data_Sheet_1.docx]

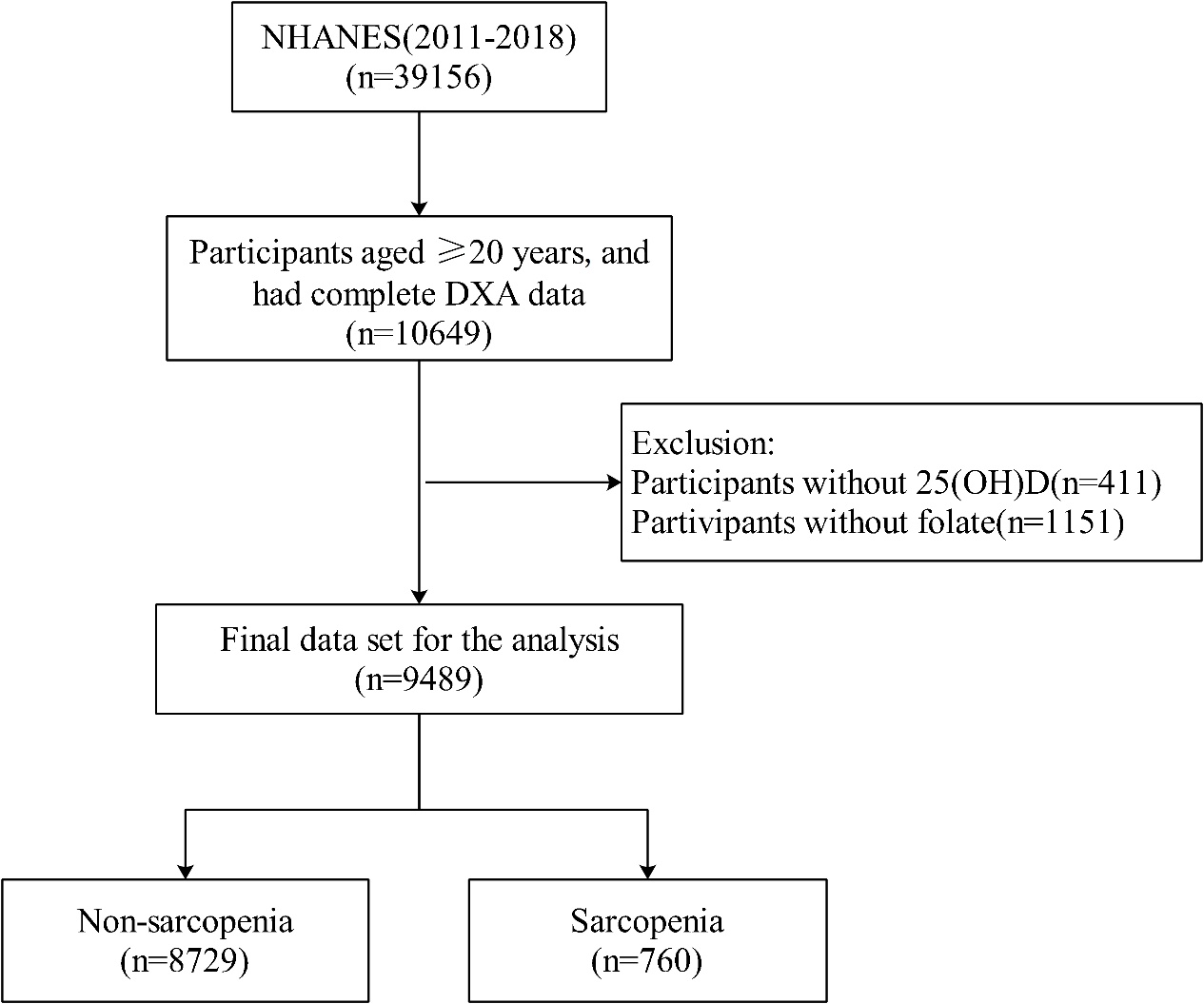


Supplemental Figure 1 Flow chart of the study participants

Supplemental Table 1 Comparison of characteristics between included and excluded persons.

| **Variables** | **Included** | **Excluded** | **P** |
| --- | --- | --- | --- |
| n | 9489 | 29667 |  |
| Age, years | 39.1±11.4 | 32.5±24.9 | <0.001 |
| Gender |  |  | 0.005 |
| Male | 4508(47.5) | 14586(49.2) |  |
| Female | 4981(52.5) | 15081(50.8) |  |
| Race |  |  | <0.001 |
| Mexican American | 1440(15.2) | 5057(17.0) |  |
| Non-Hispanic White | 990(10.4) | 3117(10.5) |  |
| Non-Hispanic Black | 3377(35.6) | 9960(33.6) |  |
| Non-Hispanic Asian | 1916(20.2) | 6584(22.2) |  |
| Other Race | 1766(18.6) | 4949(16.7) |  |
| Education level |  |  | <0.001 |
| Less than 9th grade | 581(6.1) | 1640(9.5) |  |
| 9-11th grade | 1139(12.0) | 2135(12.4) |  |
| High school graduate | 2041(21.5) | 3893(22.6) |  |
| Some college | 3118(32.9) | 5282(30.7) |  |
| College graduate or above | 2608(27.5) | 4251(24.7) |  |
| Season of examination |  |  | 0.512 |
| November 1 through April 30 | 4644(48.9) | 13747(48.5) |  |
| May 1 through October 31 | 4845(51.1) | 14571(51.5) |  |
| Smoking |  |  | <0.001 |
| No | 2031(21.4) | 4777(16.1) |  |
| Yes | 1674(17.6) | 2650(8.9) |  |
| Missing | 5784(61.0) | 22240(75.0) |  |
| Alcohol |  |  | <0.001 |
| No | 1872(19.7) | 3393(11.4) |  |
| Yes | 5692(60.0) | 7378(24.9) |  |
| Missing | 1925(20.3) | 18896(63.7) |  |
| Hypertension |  |  | <0.001 |
| Yes | 2187(23.0) | 6462(33.8) |  |
| No | 7302(77.0) | 12675(66.2) |  |
| Diabetes |  |  | <0.001 |
| Yes | 685(7.2) | 2509(8.8) |  |
| No | 8629(91.0) | 25451(89.4) |  |
| Borderline | 169(1.8) | 520(1.8) |  |
| Setting time, min | 376.0±204.1 | 391.1±166.3 | <0.001 |
| Body Mass Index，kg/m^2^ | 28.8±6.8 | 26.1±7.6 | <0.001 |
| Waist circumference，cm | 96.9±16.3 | 88.3±22.7 | <0.001 |
| Cholesterol, mmol/L | 4.9±1.0 | 4.8±0.9 | <0.001 |
| Triglyceride, mmol/L | 1.7±1.7 | 1.6±1.1 | <0.001 |
| Creatinine, umol/L | 74.9±32.6 | 76.8±31.5 | <0.001 |
| Glucose, mmol/L | 5.5±2.1 | 5.7±1.7 | <0.001 |
| Folate, nmol/L | 38.5±22.8 | 46.7±30.9 | <0.001 |
| 25(OH)D, nmol/L | 60.4±24.9 | 65.1±26.8 | <0.001 |
| Intake of energy (kcal/d) | 2229.1±1037.7 | 1966.1±966.9 | <0.001 |
| Intake of protein (gm/d) | 85.8±45.2 | 73.6±42.4 | <0.001 |
| Intake of carbohydrate (gm/d) | 265.5±131.4 | 240.4±121.9 | <0.001 |
| Intake of total fat (gm/d) | 85.3±49.0 | 76.9±45.6 | <0.001 |

Supplemental Table 2 Individual associations between 25(OH)D and folate levels with the risk of sarcopenia.

| **Variables** | **n** | **Case (%)** | **Crude model** | |  | **Adjusted model** | |
| --- | --- | --- | --- | --- | --- | --- | --- |
|  |  |  | **OR (95%CI)** | ***P*** |  | **OR (95%CI)** | ***P*** |
| **25(OH)D, nmol/L** |  |  |  |  |  |  |  |
| Per SD | 9489 | 760(8.0) | 0.86(0.79,0.93) | <0.001 |  | 0.86(0.79,0.93) | <0.001 |
| <50 | 3427 | 323(9.4) | ref |  |  | ref |  |
| 50-75 | 3756 | 289(7.7) | 0.80(0.68,0.95) | 0.009 |  | 0.69(0.58,0.82) | <0.001 |
| >75 | 2306 | 148(6.4) | 0.66(0.54,0.81) | <0.001 |  | 0.69(0.55,0.86) | 0.001 |
| *P* for trend |  |  |  | <0.001 |  |  | <0.001 |
| **Folate, nmol/L** |  |  |  |  |  |  |  |
| Per SD | 9489 | 760(8.0) | 0.97(0.89,1.05) | 0.440 |  | 0.95(0.87,1.04) | 0.296 |
| T1(<27.4) | 3154 | 274(8.7) | ref |  |  | ref |  |
| T2(27.4-42.4) | 3154 | 245(7.8) | 0.89(0.74,1.06) | 0.184 |  | 0.78(0.65,0.94) | 0.009 |
| T3(≥42.4) | 3181 | 241(7.6) | 0.86(0.72,1.03) | 0.106 |  | 0.79(0.65,0.95) | 0.011 |
| *P* for trend |  |  |  | 0.104 |  |  | 0.011 |

25(OH)D: 25-hydroxyvitamin D; The adjusted model was controlled for race and family income.


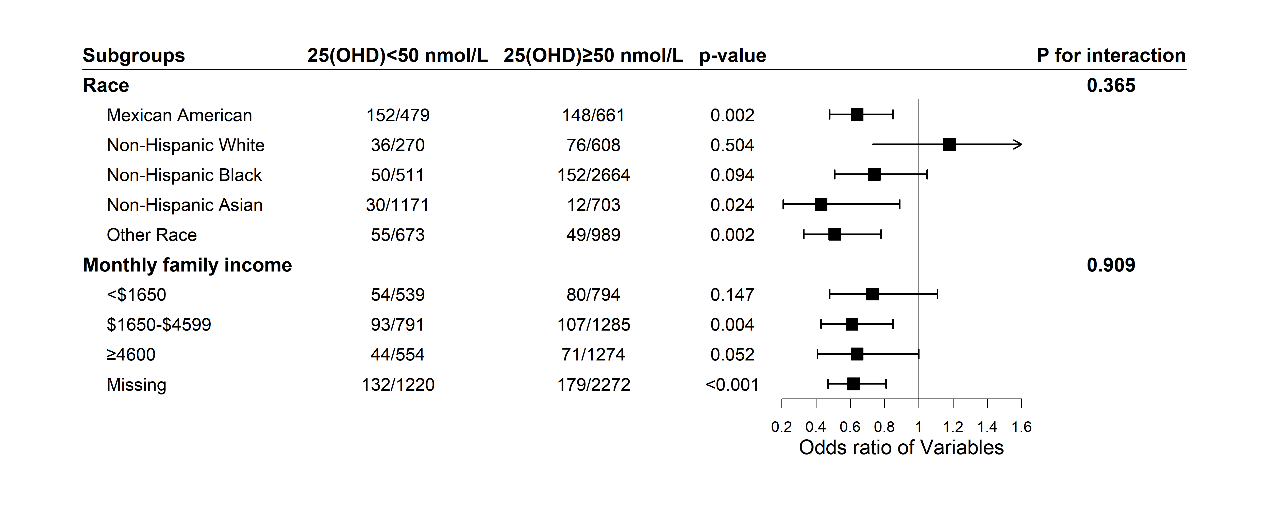


**Supplement Figure 3 Forest plots on the association of serum 25(OH)D with the risk of sarcopenia.** The adjusted model includes the following covariables: age, sex, race, education level, the season of examination, smoking status, alcohol use, sitting time, cholesterol, triglyceride, creatinine, hypertension, and diabetes, intake of energy, protein, carbohydrate, and total fat.


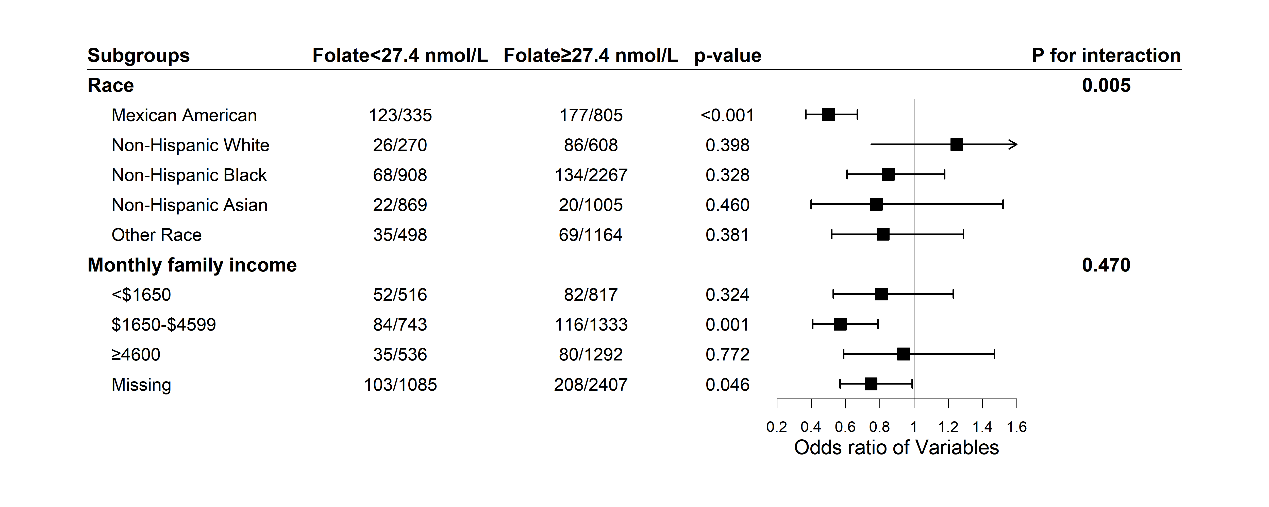


Supplement Figure 4 Forest plots on the association of serum folate with the risk of sarcopenia. The adjusted model includes the following covariables: age, sex, race, education level, the season of examination, smoking status, alcohol use, sitting time, cholesterol, triglyceride, creatinine, hypertension, and diabetes, and intake of energy, protein, carbohydrates, and total fat.

Supplemental Table 3 Individual associations between 25(OH)D and folate levels with the risk of sarcopenia among participants with body mass index less than 30 kg/m^2^.

| **Variables** | **n** | **Case (%)** | **Crude model** | |  | **Adjusted model** | |
| --- | --- | --- | --- | --- | --- | --- | --- |
|  |  |  | **OR (95%CI)** | ***P*** |  | **OR (95%CI)** | ***P*** |
| **25(OH)D, nmol/L** |  |  |  |  |  |  |  |
| Per SD | 6024 | 245(4.1) | 0.86(0.79,0.93) | 0.025 |  | 0.85(0.72,1.00) | 0.056 |
| <50 | 1938 | 94(4.9) | ref |  |  | ref |  |
| 50-75 | 2410 | 91(3.8) | 0.77(0.57,1.03) | 0.082 |  | 0.64(0.46,0.88) | 0.006 |
| >75 | 1676 | 60(3.6) | 0.73(0.52,1.01) | 0.060 |  | 0.75(0.51,1.09) | 0.135 |
| *P* for trend |  |  |  | 0.049 |  |  | <0.082 |
| **Folate, nmol/L** |  |  |  |  |  |  |  |
| Per SD | 6024 | 245(4.1) | 1.02(0.89,1.17) | 0.778 |  | 0.94(0.80,1.11) | 0.478 |
| T1(<27.4) | 1859 | 83(4.5) | ref |  |  | ref |  |
| T2(27.4-42.4) | 1991 | 73(3.7) | 0.81(0.59,1.12) | 0.210 |  | 0.63(0.45,0.89) | 0.009 |
| T3(≥42.4) | 2174 | 89(4.1) | 0.91(0.67,1.24) | 0.561 |  | 0.73(0.52,1.03) | 0.075 |
| *P* for trend |  |  |  | 0.586 |  |  | 0.085 |

25(OH)D: 25-hydroxyvitamin D; The adjusted model was controlled for age, sex, race, family income, education level, the season of examination, smoking status, alcohol use, sitting time, cholesterol, triglyceride, creatinine, hypertension, diabetes, intake of energy, protein, carbohydrate, and total fat. Additionally, serum folate levels were further adjusted for 25(OH)D levels.

Supplemental Table 4 The mediation effect between vitamin D insufficient, low folate concentration and sarcopenia among the population without chronic diseases

| **Pathways** | **Indirect effect** | **95%CI** | **Mediation** | **95%CI** | ***P*-value** |
| --- | --- | --- | --- | --- | --- |
| **Vitamin D insufficiency** |  |  |  |  |  |
| White blood cells | 2.78E-04 | -7.49E-04,0.00 | 1.68% | -1.04,0.15 | 0.520 |
| Alkaline phosphatase | 1.98E-03 | 7.47E-04,0.00 | 13.5% | -0.803,1.45 | 0.110 |
| Bilirubin | -2.33E-04 | 1.24E-04,0.00 | -1.41% | -0.150,0.18 | 0.620 |
| Gamma glutamyl transferase | 6.50E-04 | 2.81E-05,0.00 | 4.09% | -0.135,0.36 | 0.104 |
| TyG index | 1.73E-03 | 6.45E-04,0.00 | 10.0% | 0.028,0.64 | 0.028 |
| **Low folate level** |  |  |  |  |  |
| White blood cells | 7.20E-04 | -2.72E-04,0.00 | 2.80% | -0.011,0.09 | 0.140 |
| Alkaline phosphatase | 1.17E-03 | 1.56E-04,0.00 | 4.94% | 0.008,0.17 | 0.024 |
| Bilirubin | 2.02E-03 | 7.75E-04,0.00 | 8.00% | 0.030,0.26 | 0.004 |
| Gamma glutamyl transferase | 2.32E-04 | -1.48E-04,0.00 | 0.90% | -0.0073,0.04 | 0.200 |
| TyG index | 9.89E-04 | 1.96E-04,0.00 | 3.89% | 0.0067,0.14 | 0.020 |

Adjusted for age, sex, race, education level, family income, the season of examination, smoking status, alcohol use, sitting time, cholesterol, triglyceride, creatinine, hypertension, and diabetes, and intake of energy, protein, carbohydrates, and total fat.

Supplemental Table 5 The mediation effect between vitamin D insufficient, low folate concentration and sarcopenia among the population with chronic diseases

| **Pathways** | **Indirect effect** | **95%CI** | **Mediation** | **95%CI** | ***P*-value** |
| --- | --- | --- | --- | --- | --- |
| **Vitamin D insufficiency** |  |  |  |  |  |
| White blood cells | 1.16E-03 | -1.45E-03,0.00 | 2.77% | -0.042,0.10 | 0.360 |
| Alkaline phosphatase | 2.18E-03 | 3.80E-04,0.00 | 5.13% | 0.0071,0.15 | 0.020 |
| Bilirubin | 1.53E-04 | -1.17E-03,0.00 | 0.20% | -0.032,0.05 | 0.810 |
| Gamma glutamyl transferase | 3.97E-04 | -5.91E-04,0.00 | 0.79% | -0.017,0.05 | 0.440 |
| TyG index | 1.21E-03 | 4.18E-05,0.00 | 2.58% | 0.0011,0.09 | 0.040 |
| **Low folate level** |  |  |  |  |  |
| White blood cells | 2.20E-03 | -2.81E-04,0.00 | 10.2% | -0.662,1.06 | 0.212 |
| Alkaline phosphatase | 1.93E-03 | 3.95E-04,0.00 | 8.73% | -0.446,0.92 | 0.132 |
| Bilirubin | 1.40E-03 | 2.48E-04,0.00 | 6.16% | 0.442,0.76 | 0.148 |
| Gamma glutamyl transferase | 1.41E-04 | -3.05E-04,0.00 | 0.39% | -0.132,0.08 | 0.660 |
| TyG index | -3.05E-03 | -1.43E-03,0.00 | -1.02% | -0.186,0.15 | 0.600 |

Adjusted for age, sex, race, education level, family income, the season of examination, smoking status, alcohol use, sitting time, cholesterol, triglyceride, creatinine, hypertension, and diabetes, and intake of energy, protein, carbohydrates, and total fat.

Supplemental Table 6 The associations between vitamin D insufficient and relative mediator biomarkers.

|  | **β(95%CI)** | ***P*-value** |  |
| --- | --- | --- | --- |
| White blood cells | -0.05(-0.14,0.04) | 0.298 |  |
| Alkaline phosphatase | 2.27(1.30,3.24) | <0.001 |  |
| Bilirubin | 0.11(-0.11,0.34) | 0.337 |  |
| Gamma glutamyl transferase | 4.41(2.50,6.33) | <0.001 |  |
| TyG index | 0.03(0.00,0.06) | 0.024 |  |

Adjusted for age, sex, race, education level, family income, the season of examination, smoking status, alcohol use, sitting time, cholesterol, triglyceride, creatinine, hypertension, and diabetes, and intake of energy, protein, carbohydrates, and total fat.

Supplemental Table 7 The associations between Low folate level and relative mediator biomarkers.

|  | **β(95%CI)** | ***P*-value** |  |
| --- | --- | --- | --- |
| White blood cells | 0.08(-0.01,0.17) | 0.077 |  |
| Alkaline phosphatase | 1.78(0.85,2.70) | <0.001 |  |
| Bilirubin | -0.56(-0.78,-0.35) | <0.001 |  |
| Gamma glutamyl transferase | 1.56(-0.27,3.38) | 0.095 |  |
| TyG index | 0.01(-0.02,0.03) | 0.663 |  |

Adjusted for age, sex, race, education level, family income, the season of examination, smoking status, alcohol use, sitting time, cholesterol, triglyceride, creatinine, hypertension, and diabetes, and intake of energy, protein, carbohydrates, and total fat.


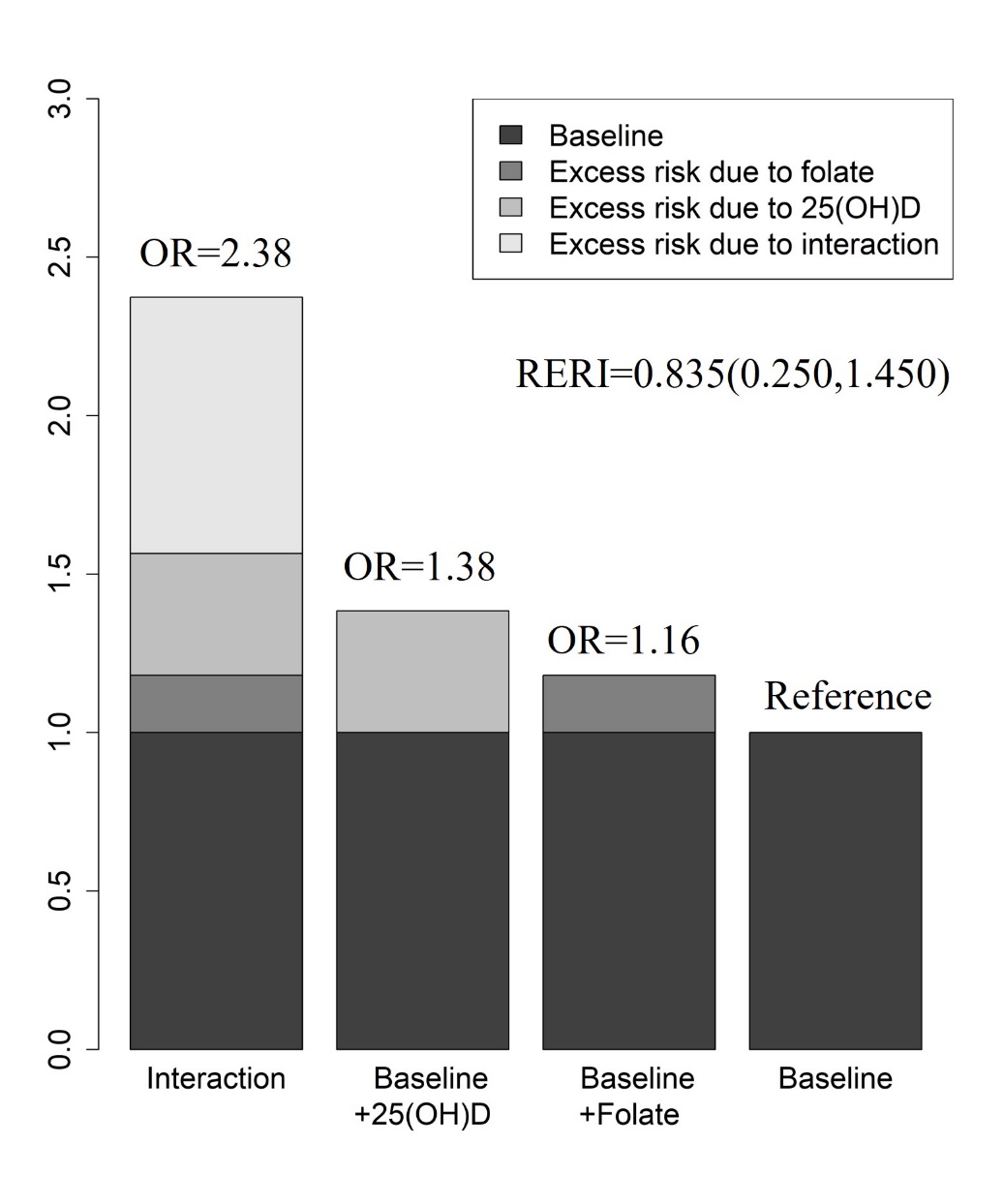


Supplement Figure 2 Joint association between 25(OH)D and folate levels with the risk of sarcopenia.
